# Dual Semi-Interpenetrating Networks of Water-Soluble Macromolecules and Supramolecular Polymer-like Chains: The Role of Component Interactions

**DOI:** 10.3390/polym16101430

**Published:** 2024-05-17

**Authors:** Anna L. Makarova, Alexander L. Kwiatkowski, Alexander I. Kuklin, Yuri M. Chesnokov, Olga E. Philippova, Andrey V. Shibaev

**Affiliations:** 1 Faculty of Physics, Lomonosov Moscow State University, 119991 Moscow, Russia; aleshina@polly.phys.msu.ru (A.L.M.); phil@polly.phys.msu.ru (O.E.P.); 2 Joint Institute for Nuclear Research, 141980 Dubna, Russia; alexander.iw.kuklin@gmail.com; 3 National Research Center, Kurchatov Institute, 123182 Moscow, Russia; chessyura@yandex.ru; 4 Chemistry Department, Karaganda E.A. Buketov University, University Street 28, Karaganda 100028, Kazakhstan

**Keywords:** self-assembly, polymer, surfactant, wormlike micelles, viscoelasticity

## Abstract

Dual networks formed by entangled polymer chains and wormlike surfactant micelles have attracted increasing interest in their application as thickeners in various fields since they combine the advantages of both polymer- and surfactant-based fluids. In particular, such polymer-surfactant mixtures are of great interest as novel hydraulic fracturing fluids with enhanced properties. In this study, we demonstrated the effect of the chemical composition of an uncharged polymer poly(vinyl alcohol) (PVA) and pH on the rheological properties and structure of its mixtures with a cationic surfactant erucyl bis(hydroxyethyl)methylammonium chloride already exploited in fracturing operations. Using a combination of several complementary techniques (rheometry, cryo-transmission electron microscopy, small-angle neutron scattering, and nuclear magnetic resonance spectroscopy), we showed that a small number of residual acetate groups (2–12.7 mol%) in PVA could significantly reduce the viscosity of the mixed system. This result was attributed to the incorporation of acetate groups in the corona of the micellar aggregates, decreasing the molecular packing parameter and thereby inducing the shortening of worm-like micelles. When these groups are removed by hydrolysis at a pH higher than 7, viscosity increases by five orders of magnitude due to the growth of worm-like micelles in length. The findings of this study create pathways for the development of dual semi-interpenetrating polymer-micellar networks, which are highly desired by the petroleum industry.

## 1. Introduction

Surfactant molecules, after reaching a critical micelle concentration, begin to self-assemble into micelles due to their amphiphilic structure. Among micelles of different shapes, worm-like surfactant micelles (WLMs) are of particular interest, since they form transient networks that impart viscoelastic properties to aqueous solutions [1,2,3]. Due to the non-covalent nature of WLMs, their solution properties are sensitive to various external factors: pH [4], temperature [5,6], presence of salts [7], other low molecular weight and polymeric substances [8,9], and so on. The viscoelastic properties of micellar solutions have practical significance and are useful for certain cosmetic applications [1,10], home care products [11,12], drug delivery [13,14,15], and oil recovery [1,16,17].

Hydraulic fracturing is a commonly used technology for enhanced oil recovery, which involves creating a network of highly conductive fractures in an area surrounding a wellbore by pumping a viscoelastic fluid with proppant particles (hydraulic fracturing fluid) [17]. Researchers have taken a novel approach to hydraulic fracturing fluids, aiming to blend the benefits of both polymer- and surfactant-based fluids [18] through the development of hybrid gels, combining polymer chains with WLMs. The interplay between polymers and surfactants governs the structure and characteristics of these fluids. By fine-tuning their interaction, researchers can create homogeneous blends keeping the shape of micellar chains.

The addition of polymers can either disrupt or keep WLMs intact, depending on their hydrophobicity. For instance, weakly hydrophobic water-soluble polymers, such as poly(vinyl methyl ether) or poly(propylene oxide) destroy WLMs [19,20,21], because the polymer chains wrap around the micelles, reducing the hydrophobic groups’ exposure to water. As a result, WLMs transform into spherical [22], ellipsoidal [19], or disc-like [23] aggregates.

By contrast, many hydrophilic polymers do not destroy WLMs, since they do not interact with them. The addition of low concentrations of such polymers does not affect the viscosity of WLM solutions [24,25,26]. But, when the concentration of hydrophilic polymer exceeds C*, a synergistic enhancement of viscosity can be observed due to entanglements between polymer and micellar chains [24]. Such dual polymer–surfactant networks, containing a transient network of WLMs and interpenetrated with polymer networks have been observed in potassium oleate/poly(vinyl alcohol) (PVA) [27] and potassium oleate/hydroxypropyl guar [28] systems.

PVA is a cheap and widely used hydrophilic polymer [29] with potential applications in hydraulic fracturing [30]. Similar to guar solutions, which are considered an industry standard in fracturing fluids [17], the viscoelasticity of PVA-based solutions can be improved by cross-linking with borate ions [30]. Furthermore, the formation of the hybrid dual network of entangled PVA chains and WLMs of surfactants can further enhance viscoelasticity [18]. At the same time, commercially available PVA always contains residual amounts of acetate groups [30], which can affect the structure of WLMs similar to the weakly hydrophobic water-soluble polymers mentioned above. The residual amount of acetate groups in PVA is influenced by the pH of the solution [30], which depends on particular fracturing conditions. To the best of our knowledge, the effect of acetate groups in PVA on dual polymer–surfactant networks has not been previously studied. This study investigated the effects of adding PVA to different acetate group content on the rheological properties and structure of mixtures with cationic surfactant EHAC in a wide range of pH.

## 2. Materials and Methods

Materials. Surfactant erucyl bis(2-hydroxyethyl)methyl ammonium chloride (EHAC) from Akzo Nobel (Amsterdam, The Netherlands), hydrotropic salt sodium salicylate (NaSal) (>99.5% purity), PVA (Mw = 22,000 g/mol) containing 12.7 mol% of acetate units from Acros and PVA (Mw = 27,000 g/mol) containing 2 mol% of acetate units from Merck (Burlington, MA, USA) were used as received.

Phase behavior and rheological properties were investigated in samples containing distilled deionised Millipore Milli-Q water. For NMR and SANS experiments, D_2_O (99.9 at% D) from AstraChem (Saint-Petersburg, Russian Federation) was used instead of H_2_O.

**Figure 1:**
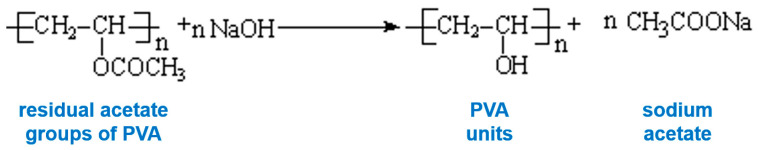
Scheme of alkaline hydrolysis of PVA acetate.

Preparation of fully hydrolyzed PVA. Firstly, a stock aqueous solution of 9.2 wt.% PVA with 2 mol% of residual acetate groups was prepared with the following method. PVA granules were added to a round-bottom flask and placed in a bath with heated silicon oil. When the temperature in the bath reached 50 °C, water was poured into the flask, and stirring with a magnetic stirrer began. The temperature evenly increased up to 95 °C for 20–25 min. Afterward, the heating was turned off, and the polymer was stirred for 30–40 min at a constant temperature. The obtained stock solution was left to cool down at room temperature while stirring. The pH of the solution was adjusted to 11 with 5 M NaOH to allow the hydrolysis of all acetate groups, which proceeds according to the reaction presented in Figure 1 [30].

Then, the solution was dialyzed in dialysis membrane tubes with a 3500 Da cutoff against 1:100 excess water for 24 h to remove sodium acetate formed due to hydrolysis and then lyophilised. The absence of residual acetate units in the as-prepared PVA was proven by ^1^H NMR.

Samples Preparation. First, aqueous stock solutions of PVA (with 12.7, 2, or 0 mol% of residual acetate groups) were prepared by dissolving PVA for 1 h at 95 °C in a round-bottom flask. Stock solutions of 5 wt.% EHAC and 11.8 wt.% NaSal in water were prepared by stirring at room temperature overnight. The final samples were prepared by mixing the appropriate stock solution amounts with a magnetic stirrer for 1–2 days. The pH of the samples was adjusted with NaOH. Before the measurements, all samples were left at room temperature for 1–2 days.

Rheology. The rheological measurements were performed with a stress-controlled rotational rheometer Physica SmartPave 102e (Anton Paar, Graz, Austria), as described elsewhere [31]. Cone-plate (diameter 40 mm, cone angle 2°) and double-gap cylindrical (mean diameter 26.4 mm, height 40 mm, gap 0.42 mm) geometries were used for viscous (with zero-shear viscosity η_0_ > 0.01 Pa·s) and liquid (η_0_ < 0.01 Pa·s) samples, respectively. The temperature was kept at 20.00 ± 0.05 °C with the Peltier elements. In steady shear experiments, the shear rate varied from 0.005 to 10 s^−1^. Oscillatory shear experiments were carried out at angular frequencies ω from 0.006 to 300 s^−1^ in a linear viscoelastic regime.

Small-angle Neutron Scattering. SANS studies were carried out in the Frank Laboratory of Neutron Physics with the YuMO spectrometer located at the IBR-2 pulsed reactor of the Joint Institute for Nuclear Research (Dubna, Russia). The samples were put into specially constructed sandwich quartz measuring cuvettes of 2 mm thickness. SANS data was collected in the range of scattering vectors q from 0.005 to 0.55 Å^−1^ [32,33]. The background scattering of KOH solutions in D_2_O at various pD values was subtracted from raw SANS data with the SAS program [34]. The obtained scattering curves were fitted by a model of a cylinder with the SasView program ver. 5.0.6 [35].

Cryogenic Transmission Electron Microscopy. Cryo-TEM experiments were performed in the bright field mode of the Titan Krios 60–300 TEM/STEM instrument (FEI) (ThermoFisher Scientific, Waltham, MA, USA) at an acceleration voltage of 300 kV. To prepare the cryo-specimens, 3 μL of the solution was deposited via the Vitrobot (FEI) [36] onto the Lacey carbon-coated side of the 300-mesh copper TEM grid (Ted Pella, Northport, NY, USA), blotted with filter paper from both sides for 3 s, plunged into liquid ethane, and transported to the microscope. For image acquisition, the microscope was equipped with a spherical aberration corrector (image corrector), a direct detection camera Falcon II (FEI), and a post-column energy filter (Gatan). A low total electron dose of less than 15 e/Å^2^ was used to avoid radiation destruction of the specimens.

^1^H Nuclear Magnetic Resonance. ^1^H NMR measurements were performed with a Bruker AV-600 spectrometer (Bruker Corporation, Billerica, MA, USA) at 25 °C. The samples were placed into standard 5 mm quartz NMR tubes (Norell, Morganton, NC, USA). Proton chemical shifts were referenced by the HOD signal at 4.70 ppm. The spectra were processed with MestReNova software (version 14.2.1-27684, MestReLab Research S.L., Santiago de Compostela, Spain).

## 3. Results and Discussion

In this article, we investigate the rheological properties and structure of hybrid self-assembled networks in aqueous solutions containing (1) a hydrophilic water-soluble polymer PVA; (2) micellar aggregates of a cationic surfactant EHAC with added hydrotropic salt NaSal. In most experiments, PVA with 12.7 mol% of residual acetate groups was used. The polymer concentration was fixed at 4 wt.% (890 monomol/L), which corresponds to the semi-diluted regime. EHAC concentration was equal to 1.25 wt.% (26 mM), and the molar ratio NaSal/EHAC was fixed at 0.35. According to the literature data, these conditions, in the absence of polymers, correspond to the formation of highly entangled viscoelastic networks of long WLMs [37]. Micelles are formed because NaSal hydrotrope anions penetrate the micellar surface, resulting in the screening of electrostatic repulsions between EHAC cationic polar heads and changes in molecular packing to the one optimal for WLMs [38]. In this work, pH varied widely from 5.5 to 11, and one-phase homogeneous solutions were obtained at all investigated conditions.

**Figure 2:**
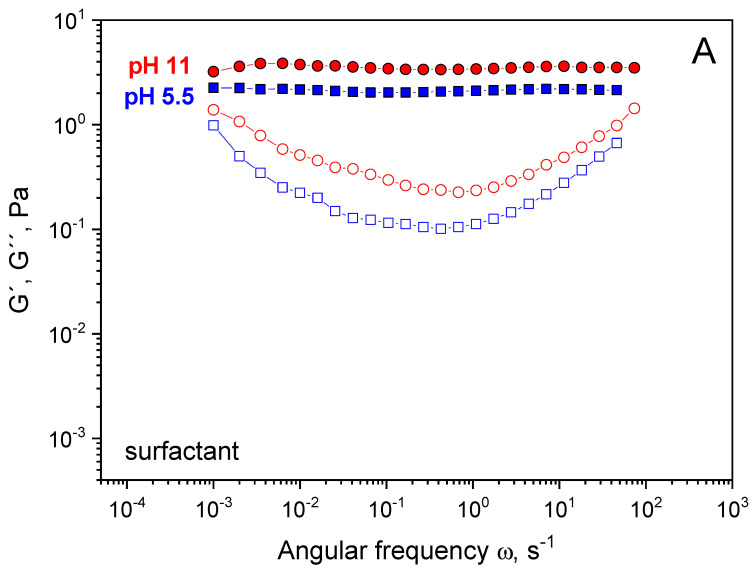

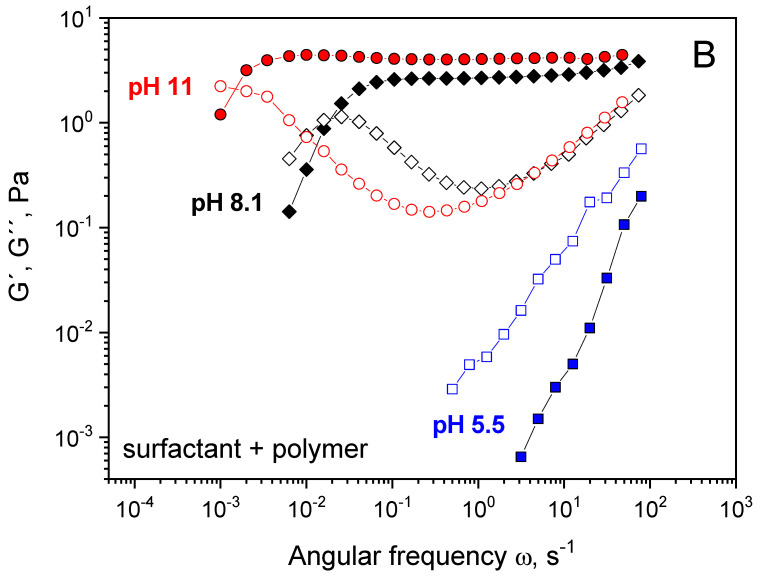
Frequency dependencies of the storage modulus G′ (full symbols) and loss modulus G″ (open symbols) for aqueous solutions containing (**A**) 26 mM EHAC and 9.1 mM NaSal at pH 5.5 (squares) and pH 11 (circles); (**B**) 26 mM EHAC, 9.1 mM NaSal and 890 monomol/L PVA with 12.7 mol% of acetate groups at pH 5.5 (squares), pH 8.1 (diamonds), and pH 11 (circles). Temperature: 20 °C.

**Figure 3:**
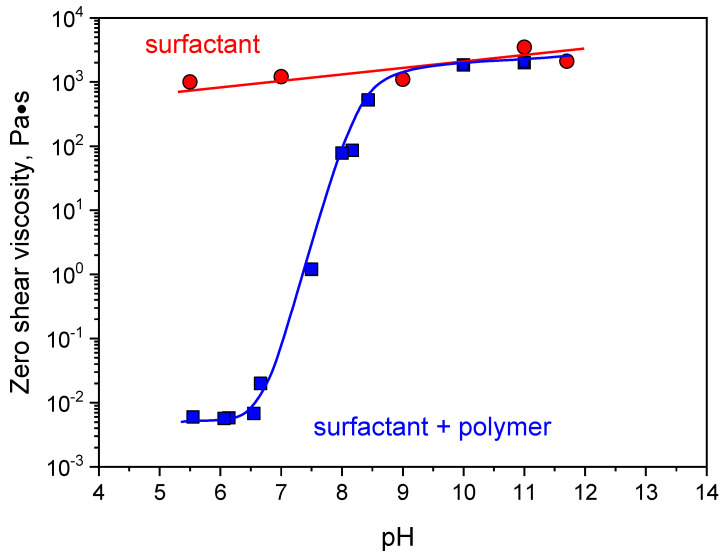
Dependencies of zero shear viscosity in pH for aqueous solutions containing 26 mM EHAC, 9.1 mM NaSal in the absence of PVA (circles) and in the presence of 890 monomol/L PVA with 12.7 mol% of acetate groups (squares).

Rheological properties First, the viscoelastic properties of surfactants and surfactant/polymer mixtures were studied. EHAC solutions in the absence of polymers show pronounced viscoelastic properties in the whole pH range from 5.5 to 11 (Figure 2A). A wide (five orders of magnitude) elastic plateau is seen at the frequency dependence of the storage modulus G′(ω), and a pronounced minimum is present at the frequency dependence of the loss modulus G″(ω). The zero-shear viscosity of the solution is six orders of magnitude higher than the viscosity of water (Figure 3). These features are characterized by highly entangled WLM networks of EHAC [37]. Rheological properties only slightly depend on pH, which is common for cationic surfactants with quaternary ammonium polar heads since they are not pH-sensitive [39]. Salicylic acid has a pK_a_ value of ca. 3 [40]; therefore, it is in its almost fully charged salt form in the studied pH range. Only a slight increase in the plateau storage modulus (Figure 2A) and viscosity (Figure 3) is observed when pH rises from 5.5 to 11, attributed to a tiny increase in ionic strength.

However, the situation differs considerably for EHAC and PVA mixtures. At low pH 5.5, the solutions show liquid-like behaviour with no viscoelasticity (Figure 2B) and low viscosity equal to 0.07 Pa·s (Figure 3), indicating a drastic change in micellar structure compared to EHAC in the absence of polymer. When pH is raised, viscoelasticity appears at around pH 7.4 and progressively develops: The elastic plateau at the dependence G′(ω) widens, and the cross-over point of G′(ω) and G″(ω) is shifted to lower frequencies, increasing relaxation time. At around pH 9, the viscosity of the mixtures reaches values characteristic of neat EHAC solutions, indicating possible restoration of WLM structures.

Thus, PVA has a significant pH-dependent effect on the viscoelastic properties of EHAC/NaSal solutions. To gain insight into the origins of this effect, structural studies by SANS and cryo-TEM were performed.

**Figure 4:**
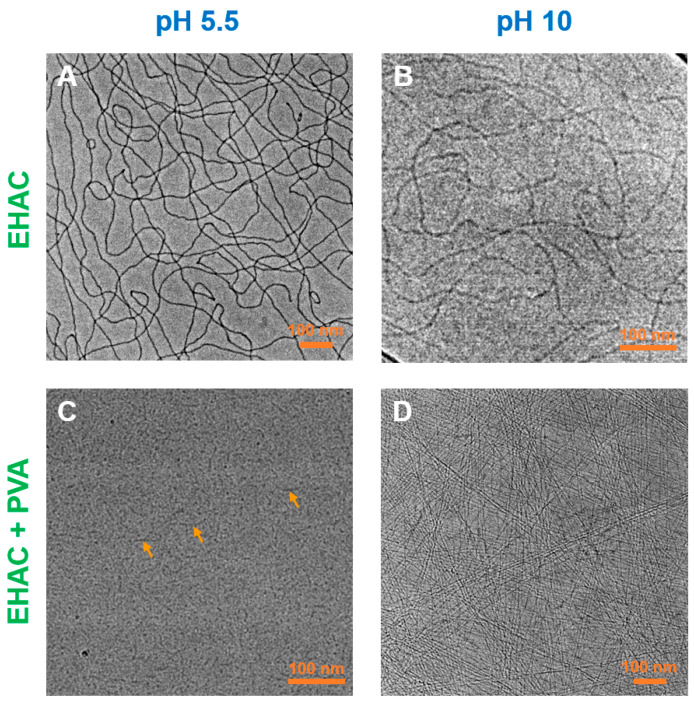
Cryo-TEM micrographs of aqueous solutions containing 26 mM EHAC and 9.1 mM NaSal: in the absence of PVA at pH 5.5 (**A**); in the absence of PVA at pH 10 (**B**); in the presence of 890 monomol/L PVA at pH 5.5 (**C**); in the presence of 890 monomol/L PVA at pH 10 (**D**). PVA contains 12.7 mol% of acetate groups.

Structure. The structure of the micellar aggregates with and without polymer was assessed by cryo-TEM. EHAC in the absence of PVA forms a network of long (micrometre length) entangled WLMs at both low and high pH (Figure 4A,B), which explains the strong viscoelasticity and high viscosity of the solutions. However, at low pH and in the presence of PVA, only small, elongated aggregates are observed (marked by arrows in Figure 4C), which do not form a network. This finding explains EHAC/PVA mixtures’ low viscosity at low pH. At the same time, a dense micellar network is restored in mixtures at high pH (Figure 4D), which is the origin of high viscoelasticity under these conditions. Note that the WLMs in this case are coupled in “bundles” [41], which are aligned parallel. Such an effect was previously observed for a system of mixed anionic/cationic WLMs (with an excess of an anionic surfactant) with PVA [42] and was explained by a partial microphase separation caused by weak repulsion between the components, leading to a local concentration of the micellar phase and the alignment of WLMs. In our case, this weak repulsion was likely absent. Indeed, there was an excess of the cationic surfactant EHAC over the anionic hydrotrope NaSal in the micelles. The literature also reports that cationic surfactants can interact with PVA [43]. However, the presence of polymers still affect the microscopic organization of WLMs.

**Figure 5:**
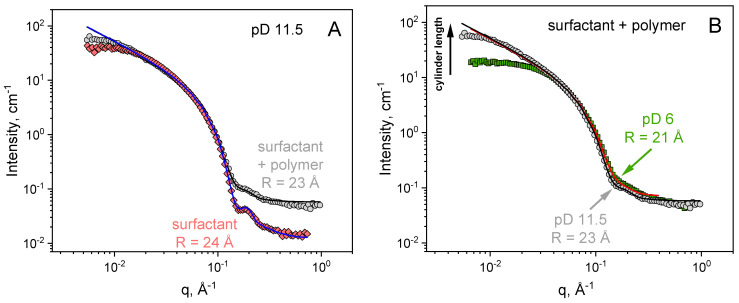
SANS scattering curves for aqueous solutions containing 26 mM EHAC and 9.1 mM NaSal: (**A**) in the absence of PVA at pD = 11.5 (diamonds, Figure 4A) and with 890 monomol/L PVA at pD = 11.5 (circles, Figure 4A,B); (**B**) with 890 monomol/L PVA at pD = 6 (squares, Figure 4B) and at pD=11.5 (circles, Figure 4A,B). Solid lines represent fits of the scattering curves with a cylinder form factor (parameter fits are summarised in Table 1). PVA contains 12.7 mol% of acetate groups.

The local structural characteristics of EHAC WLMs were investigated by SANS (Figure 5). In the absence of PVA, the scattering curves at both high pD 11.5 and low pD 6 are well-fitted by a cylinder model in a wide q range. This trend confirms that the local structure of the micelles is cylindrical and lines up with the cryo-TEM data. The radius of the cylinder is almost independent of pD and equals 24 Å, which is close to the values reported in the literature for EHAC WLMs [44]. Electrostatic repulsion between the micelles explains the small decrease in intensity at very low q compared to the cylinder form factor [45]. Indeed, their surface is charged due to excess EHAC over NaSal (the molar ratio [NaSal]/[EHAC] = 0.35).

Concerning WLM/polymer systems, the scattered intensity of neat WLMs is 1–2 orders of magnitude higher than the signal from polymers in a wide q range. WLMs significantly contribute to the scattering of the mixtures so that SANS can follow the local structure of the micelles. At high pD 11.5, the scattering profiles of the neat micelles and EHAC/PVA system are almost identical (Figure 5A) and well-fitted by a cylinder form factor. This finding indicates that the local structure of WLMs is preserved in the presence of PVA at high pD. The cross-section radius of the micelles is almost the same, but a slight increase in the radius polydispersity is observed in the presence of PVA (Table 1). Furthermore, the background is higher in the mixed system due to incoherent scattering from PVA protons.

On the other hand, a different pattern is observed at low pD (Figure 5B). The scattering curve for polymer/surfactant systems is still fitted by a cylinder form factor at intermediate and high q; however, at low q, a significant decrease in intensity is observed compared to high pD. This trend may be attributed to a decrease in the length of WLMs [46] and is consistent with cryo-TEM data showing the presence of unentangled short cylinders (Figure 4C).

Therefore, the structural investigation by cryo-TEM and SANS shows that low viscosity and the absence of viscoelasticity in the EHAC/PVA system at low pH are due to the shortening of WLMs and disruption of the entangled network. The reasons for this effect are discussed below.

**Figure 6:**
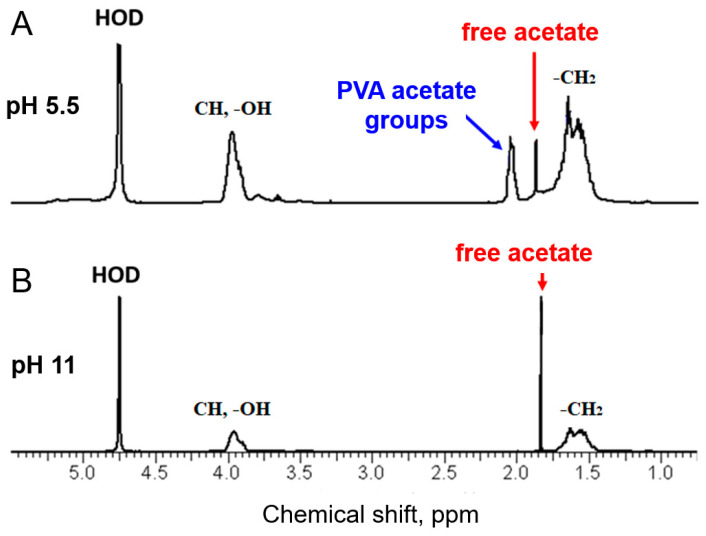
^1^H NMR spectra of 4 wt.% PVA (12.7 mol% of acetate groups) solutions in D_2_O at pH 5.5 (**A**) and pH 11 (**B**).

Molecular origin of the polymer/micellar interaction. As shown in the literature for various polymer/surfactant systems, disruption of WLMs caused by polymers is usually due to specific interactions between the components [19,20,21,22,23]. To reveal the nature of this interaction in our system and the chemical groups involved in it, ^1^H NMR spectroscopy was employed. Besides the main peaks associated with the -CH_2_- (1.4–1.75 ppm) and -CH- (3.75–4 ppm) groups of PVA [47], two additional peaks can be seen at low pH 5.5 (Figure 6A). A peak at 2.0–2.1 ppm arises from the methyl protons of the PVA residual acetate groups [48], which are present due to incomplete alkali saponification of poly(vinyl acetate) when PVA is produced [49]. NMR data shows that this PVA sample contains 12.7 mol% of residual acetate units. A narrow peak at 1.85 ppm comes from residual sodium acetate formed during the same process. At pH 11, the peak of acetate groups completely disappears (Figure 6B) due to the complete alkali hydrolysis of these groups during the preparation of polymer/surfactant mixtures according to the reaction presented in Figure 1. During this reaction, sodium acetate is produced, and its content in the solution increases.

**Figure 7:**
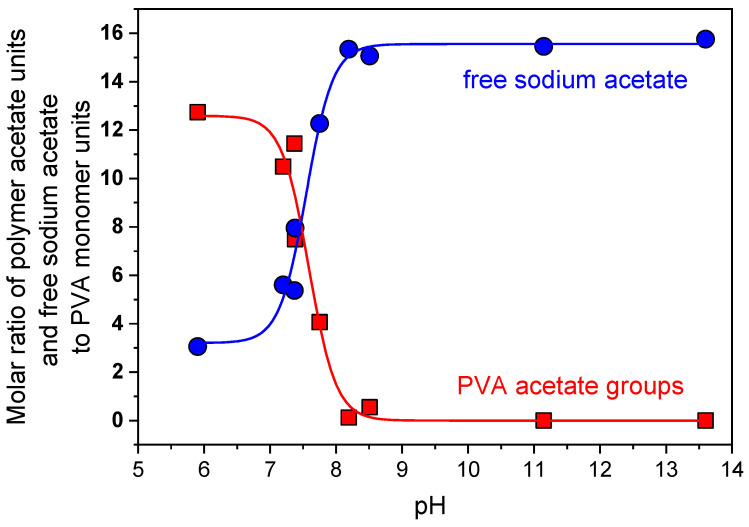
pH dependence of the molar ratio of polymer acetate units (squares) and free sodium acetate (circles) to PVA monomer units in aqueous solutions of PVA (12 mol% of acetate groups) derived from NMR data.

pH dependence on the number of PVA residual acetate groups and “free” sodium acetate in PVA/EHAC mixtures is presented in Figure 7. Hydrolysis starts immediately at alkali conditions (above pH 7) and finishes at pH 8.1. This pH range fully coincides with the range where the viscosity increase in PVA/EHAC mixtures is observed (Figure 3). Therefore, it can be concluded that residual acetate groups of PVA are responsible for the interaction with WLMs and their shortening at low pH levels.

**Figure 8:**
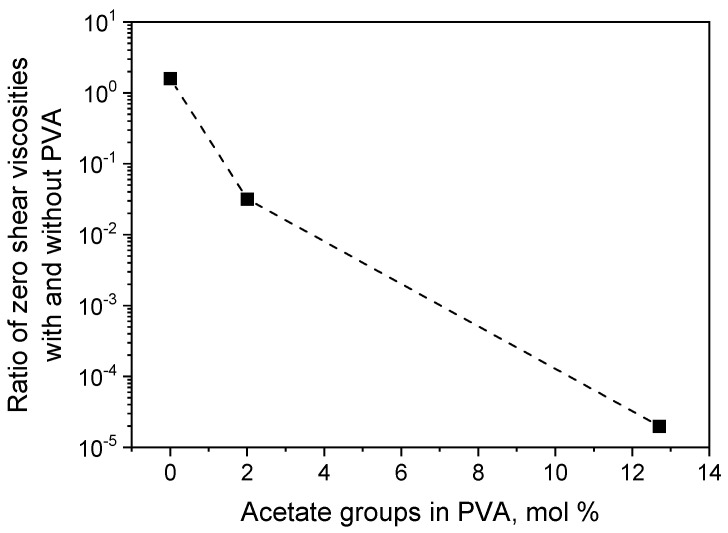
Ratios of zero-shear viscosities of micellar solutions (26 mM EHAC and 9.1 mM NaSal) with 890 monomol/L PVA and without polymer vs. amount of residual acetate groups in PVA at pH 5.5. Temperature: 200 °C.

To further prove this conclusion, we investigated the effect of residual acetate group content in PVA on the viscosity of EHAC/NaSal/PVA solutions at low pH (Figure 8). The viscosity of polymer/micellar solutions drastically decreases with an increase in acetate unit content, indicating that acetate units are responsible for disrupting the WLM network.

**Figure 9:**
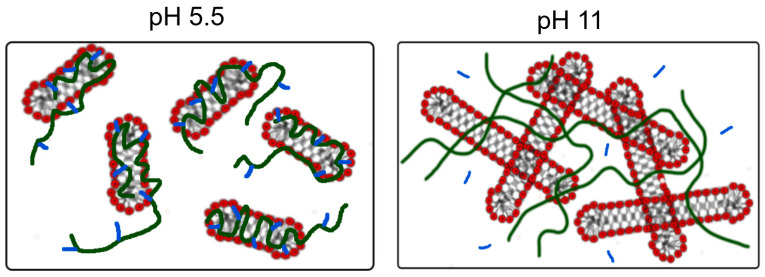
Schematic representation of the interaction between PVA and EHAC WLMs at low and high pH. Surfactant molecules are represented by red and grey. PVA molecules are indicated by green lines. Residual PVA acetate units and sodium acetate formed due to hydrolysis are indicated by blue short lines.

The reason for this behaviour is that acetate groups are slightly hydrophobic [50] and can interact with the micellar surface. However, they cannot penetrate deep into the micellar hydrophobic core due to their small size. Consequently, they probably reside in the micellar corona and change the molecular packing of the surfactant molecules, increasing the average distance between their heads and decreasing the molecular packing parameter, leading to the shortening of the micelles. This effect is schematically depicted in Figure 9. It should be noted that the influence of small acetate groups on WLM geometry differs from the effect of long alkyl side chains of hydrophobically modified polymers [51,52], which do not disrupt or shorten WLMs. This finding is due to long alkyl groups penetrating deeper into the micellar core, possibly even strengthening the hydrophobic interactions between the surfactant tails.

When pH is raised, residual acetate groups are hydrolysed and PVA no longer interacts with WLMs, so their high length is restored. Consequently, a common entangled network of WLMs and polymer molecules is formed (Figure 7). It should be noted that if the pH of the PVA/EHAC mixture is lowered from 11 to 5.5, no decrease in viscosity is observed because fully saponified PVA no longer interacts with WLMs.

**Figure 10:**
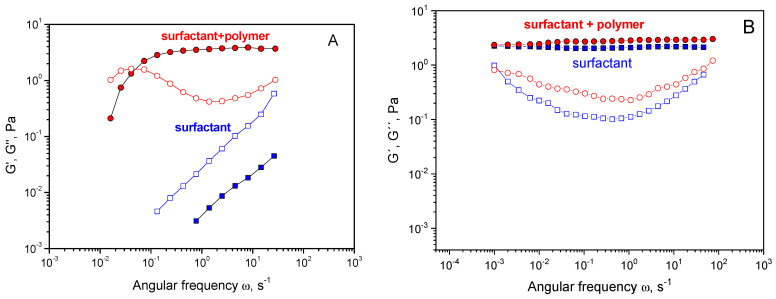
Frequency dependencies of the storage modulus G° (full symbols) and loss modulus G″ (open symbols) for aqueous solutions containing (**A**) 6.2 mM EHAC and 2.2 mM NaSal; (**B**) 26 mM EHAC and 9.1 mM NaSal in the absence of PVA (squares) and in the presence of 890 monomol/L fully hydrolysed PVA (circles) at pH 5.5. Temperature: 20 °C.

An investigation of polymer/WLM viscoelastic properties revealed the formation of an entangled semi-interpenetrating network of WLMs and PVA. For this purpose, fully hydrolysed PVA is mixed with WLMs, which should not interact with them due to the absence of acetate groups. At low EHAC concentrations (6.2 mM), where the micellar network is at the onset of formation, the addition of fully hydrolysed PVA results in a drastic change in rheological properties (Figure 10A). For micelles without PVA, no viscoelasticity was observed because an entangled WLM network was not formed at this concentration. In the presence of PVA, clear viscoelasticity developed with a plateau at the G′(w) dependence and a cross-over point between G′(w) and G″(w). This finding can be attributed to the appearance of entanglements between micelles and PVA macromolecules, resulting in the formation of a common network. At high EHAC concentrations (26 mM), the addition of fully hydrolysed PVA slightly increases the plateau storage modulus due to the formation of additional entanglements between polymer and micellar chains as well as entanglements already formed between EHAC micelles at this concentration. Therefore, at 26 mM EHAC, WLMs already form an entangled network. PVA chains are incorporated into this network by forming additional entanglements with the micelles.

## 4. Conclusions

This paper reports on our investigation of the effect of polymer–surfactant interactions on the structure and rheological properties of dual networks formed by interlaced polymers and micellar chains of nonionic polymer PVA and cationic surfactant EHAC. Using cryo-TEM and SANS, we demonstrate that polymers induce the shortening of micelles at pH levels below 7, which was explained by incorporating residual acetate groups of polymers into the micelles. This effect disappears at a higher pH due to the removal of these groups because of hydrolysis, which was proved by NMR data. As a result, the length of the micelles increases tremendously, resulting in viscosity enhancement by five orders of magnitude. These results are important for properly combining certain polymer and surfactant components as thickening agents in various formulations.

## Figures and Tables

**Table 1:** Parameter fits of SANS scattering curves in Figure 4 using a cylinder form factor.

pD	EHAC Concentration, mM	PVA Concentration, monomol/L	Radius of Cylinder R, Å	Radius Polydispersity ΔR/R
6	26		24	0.15
6	26	890	21	0.25
11.5	26		24	0.15
11.5	26	890	23	0.2

## Data Availability

The data presented in this study are available at https://drive.google.com/ upon request.
